# Otolaryngology Involvement in Emergency Care for Dizziness During the 2024 Korean Resident Physician Workforce Disruption and Subsequent Return: A Single-Center Retrospective Study

**DOI:** 10.3390/healthcare14162618

**Published:** 2026-08-19

**Authors:** Jung Min Kim, Hye Ok Kim, Jae Min Lee, Dong Gu Hur, Seung Geun Yeo

**Affiliations:** 1 Department of Otorhinolaryngology, Head and Neck Surgery, School of Medicine, Kyung Hee University Hospital, 23 Kyung Hee Dae-ro, Dongdaemun-gu, Seoul 02447, Republic of Korea; kpax1727@naver.com (J.M.K.); sunjaesa@hanmail.net (J.M.L.); mdhur@hanmail.net (D.G.H.); 2 Clinical Research Institute, Kyung Hee University Medical Center, Seoul 02447, Republic of Korea; hyeokkim@khu.ac.kr

**Keywords:** dizziness, emergency department, care pathways, health workforce, specialist consultation, diagnostic imaging, diagnostic yield, health services research

## Abstract

**Background/Objectives:** We used dizziness as a tracer condition to describe documented emergency-care changes during resident physician workforce disruption and return, and whether greater diagnostic intensity was accompanied by greater diagnostic yield. **Methods:** This single-center retrospective observational study compared two 13-month periods: no-resident (February 2024–February 2025) and resident-return (March 2025–March 2026). Registry data for all 1652 eligible emergency department (ED) dizziness visits provided primary treating department, an administrative department-of-record field, disposition, and length of stay. Detailed chart review was limited to patients with a formally documented otorhinolaryngology (ENT) consultation. Monthly staffing and on-call coverage came from duty rosters, and brain MRI reports were classified by clinical relevance. No causal inference was intended. **Results:** Dizziness represented a similar proportion of ED visits across periods (5.9% vs. 5.4%; rate ratio [RR], 0.91; *p* = 0.061). Formally documented ENT consultation increased from 26/667 (3.9%) to 186/985 (18.9%; RR, 4.84; *p* < 0.001), although ENT on-call coverage was recorded throughout and combined attending/fellow staffing declined from nine to six. Neurology department-of-record decreased from 24.7% to 13.0% and otolaryngology increased from 3.3% to 17.7% (both *p* < 0.001), while overall admission remained stable (24.1% vs. 23.9%; *p* = 0.896). Crude hospital length of stay decreased from 4.68 to 3.51 days (*p* = 0.002), but not after otolaryngology admissions were excluded (4.68 vs. 5.54 days; *p* = 0.208), indicating a case-mix effect. Among ENT-consulted patients, MRI utilization increased from 57.7% to 95.7% (*p* < 0.001); 1/192 classifiable studies showed an acute central lesion (0.5%) and 7 (3.6%) incidental lesions. Dizziness-specific revisits did not differ at 72 h or 30 days. **Conclusions:** Documented consultation, imaging, and administrative departmental attribution changed markedly, whereas dizziness burden, overall admission, and short-term revisits remained stable. Differential documentation plausibly explains a substantial part of the consultation difference, which measures documented rather than actual specialist involvement, and greater imaging intensity was not accompanied by greater acute diagnostic yield. These descriptive findings do not establish that care became better, safer, or more accurate.

## 1. Introduction

In February 2024, the Korean government announced an immediate 65% increase in the national medical school admission quota. In protest, more than 90% of the country’s approximately 13,000 resident physicians resigned within weeks, producing a near-total and prolonged absence of trainees at teaching hospitals nationwide [1]. Because Korean tertiary hospitals have traditionally relied on residents for much of the emergency department (ED) triage, bedside specialty examination, documentation, and interdepartmental coordination, this disruption—and the phased return of trainees that followed—created an unusual setting in which to observe how abrupt changes in workforce availability are reflected in the delivery of emergency care [1,2,3,4].

Studies of this disruption, and of comparable events elsewhere, have reported reductions in hospital admissions, length of stay, expenditure, and elective or major operative volumes, generally without a consistent corresponding signal in short-term mortality [1,2,3,4]. This pattern is not unique to Korea: a systematic review and meta-analysis of health care strikes across multiple countries and professions likewise found no significant difference in in-hospital mortality between strike and non-strike periods [5]. A parallel literature on the annual turnover of trainees in teaching hospitals has likewise identified changes in efficiency and, in higher-quality studies, in mortality at the time of changeover, although heterogeneity across studies precludes firm conclusions about the magnitude of risk [6]. Taken together, these findings are consistent with a long-standing observation in health services research: structural change in a care system is often expressed first in the processes of care—how patients are triaged, referred, investigated, and admitted—rather than in the outcome measures that are most commonly reported [7]. Process measures, however, occupy an ambiguous position, because a change in the intensity or completeness of documented care activity is not by itself evidence that care became better, safer, or more accurate.

Observing process change requires a clinical presentation whose management depends on coordinated input from several services rather than on a single decision point—the logic of the tracer condition, in which a common, well-defined clinical problem is used to make the functioning of a health care system visible [8]. Dizziness is well suited to this role. It accounts for roughly 3–4% of ED visits worldwide and generates substantial and rising resource use [9,10,11]. It is also diagnostically demanding: presentations range from self-limited peripheral vestibular disorders to central causes including posterior circulation stroke, which accounts for a small but consequential minority of ED dizziness presentations and is disproportionately represented among missed cerebrovascular diagnoses [12,13,14]. Accurate discrimination depends on serial reassessment, structured bedside oculomotor examination, and—when indicated—neuroimaging and specialist consultation [15,16,17]. Yet the thresholds governing these steps are neither uniform nor settled. Whether patients with acute dizziness should routinely receive neuro-otologic or neurologic consultation rather than bedside evaluation by the treating emergency physician alone remains actively debated [18]; neuroimaging utilization varies substantially by care setting [19]; and the diagnostic yield of MRI in this population is low relative to the frequency with which it is obtained [20]. Because dizziness care sits at the intersection of variable consultation thresholds, discretionary imaging decisions, and interdepartmental coordination, it may be unusually sensitive to disruption of the workforce that ordinarily performs that coordination. We therefore treat dizziness not as a disease of interest in itself, but as a symptom-based tracer condition through which changes in specialist access and diagnostic workflow can be observed over time. Korean single-center series have described the epidemiology and ED disposition of dizziness patients under ordinary staffing conditions, providing context for the present setting [21,22].

Most analyses of the 2024 Korean disruption have examined system-wide, ED-wide, or surgical endpoints; how the disruption and the subsequent phased restoration of trainees coincided with the documented care pathway for a specific, common, multidisciplinary ED symptom has received comparatively little attention. This gap matters for a reason beyond description. If restoration of the workforce is accompanied by changes in consultation, imaging, and departmental attribution, the substantive question is not whether documented activity changed, but whether those changes are accompanied by evidence of greater diagnostic return or a more favorable short-term clinical course. Distinguishing care intensity from care quality is a recurring problem in health services research, and greater service activity is not invariably beneficial [7,23].

Accordingly, this single-center retrospective observational study describes temporal changes in formally documented ENT consultation, diagnostic imaging, diagnostic labeling, departmental attribution, and disposition among ED visits for dizziness across a no-resident period and a subsequent resident-return period at a Korean tertiary hospital. To characterize the exposure more concretely than a calendar boundary permits, we additionally assembled month-level counts of ENT residents, attending physicians, and clinical fellows from departmental duty rosters. To examine whether greater diagnostic intensity corresponded to greater diagnostic return, we reviewed all brain MRI reports and classified them according to clinical relevance. Finally, we examined short-term dizziness-specific ED revisits as a partial indicator of early clinical course. We hypothesized that the relative burden of dizziness would remain similar across periods, whereas documented care pathways would differ. Given the observational design and concurrent changes in hospital operations, staffing, and documentation practices, associations with resident staffing were interpreted descriptively rather than causally.

## 2. Materials and Methods

### 2.1. Study Design and Setting

This single-center retrospective observational study was conducted at Kyung Hee University Hospital, a tertiary referral hospital in Seoul, South Korea. We reviewed ED visits for dizziness or vertigo from 20 February 2024 to 31 March 2026. During the study period, ED operations were reduced following the resident workforce disruption and subsequently returned toward routine capacity. Contracted non-training physicians were used to support emergency coverage during the disruption period.

In the institutional medical records department and the department of radiology, we additionally enquired whether any formal changes had been made to brain MRI ordering pathways, to specialty consultation procedures, or to the electronic consultation recording system between the two study periods. No such change was identified. The principal documented institutional change across the study window was the reduction and subsequent recovery of emergency department operating capacity described above.

### 2.2. Study Periods and ENT Physician Staffing

For the primary analysis, the study window was divided into two consecutive 13-month periods: a no-resident period (February 2024 to February 2025) and a resident-return period (March 2025 to March 2026). These labels were used for the primary calendar-based comparison; actual resident availability varied within the two periods.

Monthly numbers of ENT residents, attending physicians, and clinical fellows were obtained from departmental duty rosters throughout the study period. The rosters were also reviewed to determine whether ENT emergency on-call coverage was available each month. February 2024 was a partial month beginning on 20 February 2024 and included two senior residents who remained on duty immediately before the mass resignation.

### 2.3. Data Sources and Case Identification

Aggregate monthly counts of total ED visits and ED visits with dizziness or vertigo recorded as the chief complaint were obtained from the institutional medical records department. Eligible dizziness visits were identified from the ED registry and verified by chart review. Visits were excluded if dizziness or vertigo was not the actual chief complaint, if the presenting symptoms were clearly attributable to acute trauma, or if key data required for the analysis were unavailable.

Patient-level registry data were available for all eligible dizziness visits and included age, sex, ED arrival and departure times, primary treating department, ED disposition, and, for admitted patients, hospital discharge time. For patients with a formally documented ENT consultation, additional clinical data were extracted from the electronic medical record, including ED triage records, consultation notes, admission records, nursing records, and radiologic reports. These additional variables included mode of arrival, presenting symptoms, time from symptom onset to ED arrival, relevant comorbidities, ENT and other specialty consultations, brain MRI use, documented ENT diagnosis, and disposition. Records were linked using patient identifier and visit date.

### 2.4. Measures and Definitions

The prespecified primary pathway measure was the proportion of dizziness visits with a formally documented ENT consultation; all other measures were secondary or exploratory. System-level measures included the proportion of dizziness visits among all ED visits, the proportion of dizziness visits with a formally documented ENT consultation, overall hospital admission among dizziness visits, and the distribution of admitting department among admitted patients. ED length of stay was calculated from ED arrival to ED departure for all dizziness visits. Among admitted patients, hospital length of stay was calculated from ED departure to hospital discharge. Patient-level clinical measures were evaluated among patients who received an ENT consultation. A formally documented ENT consultation was defined as a consultation entered in the electronic medical record; informal discussion between the ED and ENT service without a consultation record was not captured. Because consultation entries were ordinarily generated by resident physicians, this measure is documentation-dependent and its ascertainment is not independent of the staffing conditions being compared. Throughout this report, “formally documented ENT consultation” therefore denotes the presence of a consultation record, and “ENT-consulted patients” denotes patients for whom such a record exists. Neither term denotes actual ENT clinical involvement, which could not be ascertained from these data.

Brain MRI was ordered by the treating emergency physician in all cases, and no institutional protocol governed neuroimaging in acute dizziness during either period. The electronic order did not include a structured indication field, and indications for imaging could not therefore be retrieved retrospectively. Descriptively, during the no-resident period imaging decisions were made at the discretion of the treating emergency physician. After resident-return, consultations were performed by trainees under supervision, and in the absence of a standardized and locally validated bedside oculomotor protocol the consulting service adopted a low threshold for requesting brain MRI: imaging was generally requested unless the presentation was unambiguously peripheral or the patient declined after explanation. This account describes departmental practice as reported by the clinicians involved and could not be verified at the level of the individual patient.

Standardized bedside vestibular examination protocols, including the HINTS battery, were not applied or recorded as a protocolized assessment in either period. Spontaneous and positional nystagmus and the head impulse (head thrust) test were documented in the consultation record at the discretion of the examiner.

Brain MRI findings were classified according to the final radiology report as: (1) acute central lesion, including acute infarction or intracranial hemorrhage; (2) incidental lesion unrelated to the presenting symptom but warranting follow-up or further evaluation; (3) chronic or nonspecific finding, including chronic infarction, white matter changes, cerebral microbleeds, or age-related atrophy; or (4) normal study [24,25]. Reports that could not be classified were excluded from this analysis.

The documented ENT diagnosis was defined as the diagnosis recorded by the consulting ENT service. Because no independent diagnostic adjudication or retrospective application of standardized diagnostic criteria was performed, diagnoses were analyzed as documented rather than as validated final diagnoses.

For patients with a formally documented ENT consultation, admission to any hospital department was recorded, and admitted patients were further identified as admitted under ENT or under another department; interfacility transfer was recorded separately. A dizziness-specific ED revisit was defined as a subsequent ED visit by the same patient with dizziness recorded as the chief complaint within 72 h or 30 days of the index visit. Index visits without sufficient follow-up within the study window were excluded from the corresponding revisit analysis.

Primary treating department was the department of record for the ED visit in the institutional registry, available for all dizziness visits. For admitted patients, the admitting department was defined by the same field. This is an administrative attribution field and does not denote a formally documented specialty consultation. Hospital length of stay was additionally examined by admitting department to assess whether differences in the overall comparison reflected changes in the composition of admitted patients.

### 2.5. Statistical Analysis

Categorical variables are presented as frequencies and percentages and compared using the chi-square test or Fisher’s exact test, as appropriate; where multiple sparse categories were compared, Fisher’s exact test was used. Continuous variables are presented as median and interquartile range (IQR) and compared using the Mann–Whitney U test or the Kruskal–Wallis test. Overall admission and length-of-stay outcomes were compared between the two study periods using all eligible dizziness visits, with hospital length of stay restricted to admitted patients. System-level effects between the two prespecified periods are expressed as rate ratios (RRs) or odds ratios (ORs) with 95% confidence intervals (CIs). Benjamini–Hochberg false discovery rate q-values were calculated within the family of patient-level clinical characteristics compared between periods [26]. No formal multiplicity adjustment was applied to the remaining comparisons, which are reported as unadjusted *p*-values and interpreted as exploratory.

**Figure 1:**
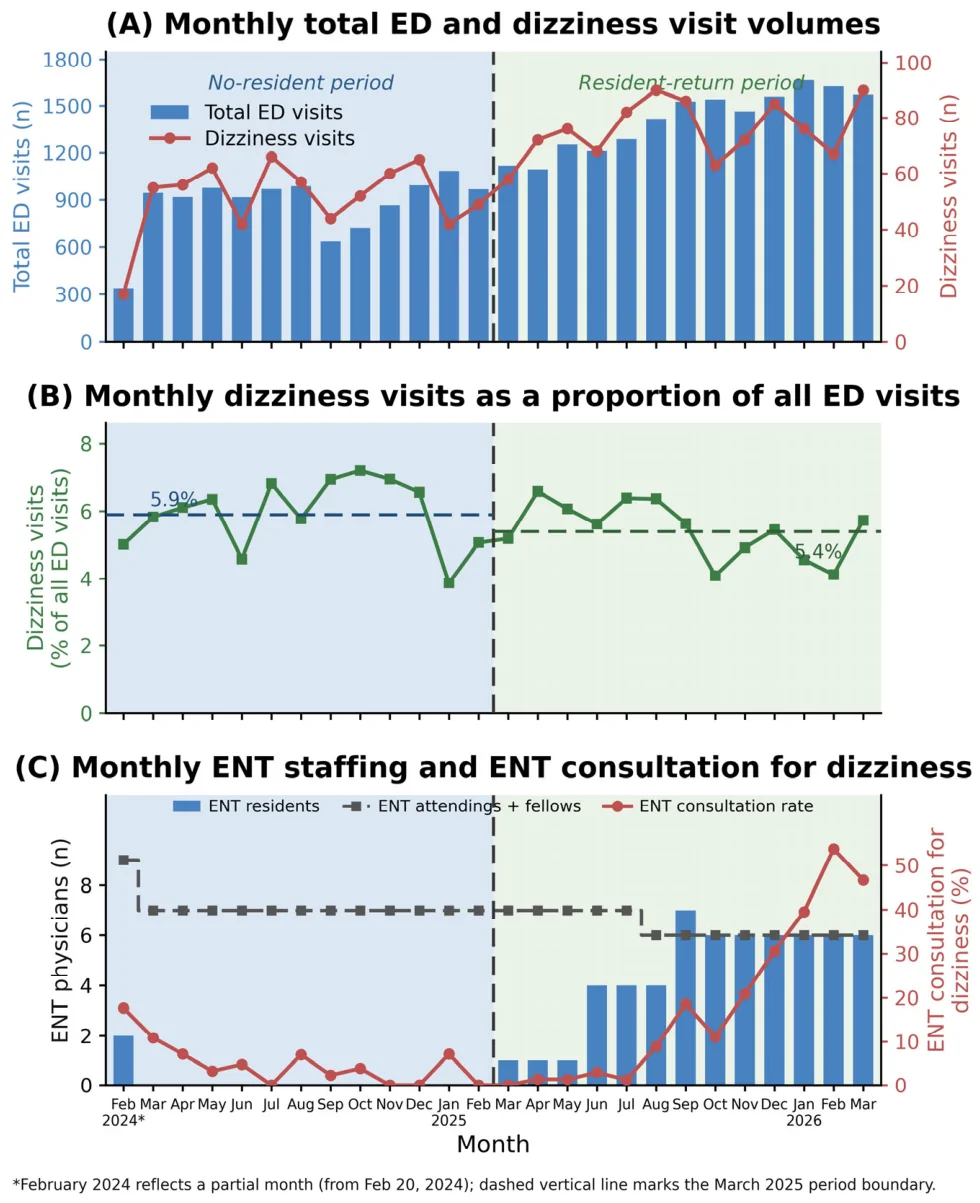
Monthly emergency department (ED) activity, dizziness visits, and otorhinolaryngology (ENT) physician staffing across the study window (February 2024–March 2026). (**A**) Monthly total ED visits (left axis, bars) and dizziness visits (right axis, line). Shaded regions indicate the no-resident period (February 2024–February 2025) and resident-return period (March 2025–March 2026); the vertical dashed line marks the March 2025 period boundary. (**B**) Monthly dizziness visits as a proportion of all ED visits; horizontal dashed lines indicate the period-level proportions (5.9% and 5.4%). (**C**) Monthly ENT resident headcount (left axis, bars), combined ENT attending and clinical fellow headcount (left axis, dashed line), and the proportion of dizziness visits with a formally documented ENT consultation (right axis, line). February 2024 represents a partial month beginning on 20 February 2024.

Monthly data were used to describe temporal patterns in staffing and care pathways (Figure 1). Pathway measures were also summarized across the five staffing phases shown in Table 1. In an exploratory analysis, we fitted a Poisson regression model of the monthly number of formally documented ENT consultations for dizziness on the monthly number of ENT residents, using the monthly number of dizziness visits as an offset. The model was examined both without adjustment and with adjustment for linear calendar time.

Overdispersion was assessed using the Pearson chi-square statistic divided by the residual degrees of freedom and by likelihood-ratio comparison against a negative binomial model. Because overdispersion was present, the negative binomial model is reported as the primary exploratory specification, and quasi-Poisson estimates—Poisson point estimates with standard errors scaled by the square root of the Pearson dispersion statistic—are reported as a sensitivity analysis. The correlation between monthly resident headcount and calendar month was also examined.

These exploratory analyses were used to characterize temporal associations rather than to estimate causal effects.

To assess whether the between-period shift in primary treating department differed by time of arrival, visits were classified as on-hours (08:00–18:00 on weekdays) or off-hours (18:00–08:00 on weekdays and at any time on Saturdays and Sundays). Exploratory logistic regression models included study period, time stratum, and their interaction, with otolaryngology and neurology department-of-record status examined separately (Table S4).

Univariable and multivariable logistic regression identifying factors associated with admission among ENT-consulted patients, together with the exploratory disease-specific and BPPV-subtype analyses, are presented in the Supplementary Materials (Tables S1–S3). A two-sided *p* < 0.05 was considered significant. Missing values were not imputed; analyses used available cases, and multivariable regression was restricted to complete cases. Analyses were performed in Python 3.11 (pandas 3.0.3, SciPy 1.17.1, statsmodels 0.14.6; all open-source software). The study is reported in accordance with the Strengthening the Reporting of Observational Studies in Epidemiology (STROBE) statement [27].

### 2.6. Ethics Statement

This study was approved by the Institutional Review Board of Kyung Hee University Hospital (KHUH IRB No. 2023-04-060; approved 28 April 2023), and the requirement for informed consent was waived owing to the retrospective design. The approval period was subsequently extended (approved 27 May 2026) to cover the present study period.

## 3. Results

### 3.1. ED Activity, Dizziness Burden, and ENT Staffing

There were 11,314 total ED visits and 667 dizziness visits during the no-resident period, and 18,325 total ED visits and 985 dizziness visits during the resident-return period (Table 1). Total ED and dizziness visit volumes increased progressively across the study window, with monthly total ED visits rising from a low of 635 (September 2024) to a peak of 1665 (January 2026) (Figure 1A). The proportion of ED visits attributable to dizziness was similar between periods (5.9% vs. 5.4%; RR, 0.91; 95% CI, 0.83–1.00; *p* = 0.061) and remained within a comparable range throughout, without a discrete change at the period boundary (Figure 1B).

Month-level duty rosters showed that resident availability changed in stages rather than at the calendar boundary (Table 1, Figure 1C). No ENT residents were on duty from March 2024 through February 2025; one resident returned in March 2025, four from June 2025, and six to seven from September 2025. Over the same window the combined number of ENT attending physicians and clinical fellows declined from nine to six. ENT emergency on-call coverage was recorded throughout the study period. Across the five consecutive staffing phases, dizziness accounted for a similar proportion of ED visits (4.9–6.1%), whereas the proportion of dizziness visits with a formally documented ENT consultation was 3.5% when no residents were on duty, 1.0% and 4.6% during the first and second return phases, and 32.1% in the final phase.

In an exploratory model with the monthly number of dizziness visits as offset, the Poisson specification indicated that each additional ENT resident was associated with a higher rate of formally documented ENT consultation for dizziness (incidence rate ratio [IRR], 1.48; 95% CI, 1.39–1.59). Overdispersion was substantial: the Pearson chi-square divided by residual degrees of freedom was 4.51 without adjustment and 5.65 after adjustment for linear calendar time, and the negative binomial dispersion parameter was 0.57 (likelihood-ratio test versus Poisson, *p* < 0.001). The Poisson confidence intervals are therefore narrower than warranted. In the negative binomial model, the association persisted (IRR, 1.44; 95% CI, 1.26–1.63; *p* < 0.001), as it did under quasi-Poisson estimation (IRR, 1.48; 95% CI, 1.28–1.72; *p* < 0.001). After adjustment for linear calendar time the estimate was not robust to model specification: the quasi-Poisson estimate was no longer significant (IRR, 1.27; 95% CI, 0.94–1.71; *p* = 0.119), whereas the negative binomial estimate exceeded the unadjusted one (IRR, 1.88; 95% CI, 1.37–2.60). Monthly resident headcount and calendar month were highly correlated (r = 0.85), and staffing cannot be separated from secular trend in these data (Table S6). These analyses are exploratory and descriptive; staffing was not randomly assigned and several institutional changes occurred concurrently.

### 3.2. Formally Documented ENT Consultation and Short-Term Revisit

Formally documented ENT consultation differed between periods (Table 2). A formally documented ENT consultation was recorded for 26 of 667 dizziness visits (3.9%) in the no-resident period and 186 of 985 (18.9%) in the resident-return period (RR, 4.84; 95% CI, 3.25–7.22; *p* < 0.001). As a proportion of all ED visits, dizziness-related ENT consultations increased from 0.23% to 1.02% (RR, 4.42; 95% CI, 2.93–6.65; *p* < 0.001). Despite this increase in formally documented ENT consultation, the overall proportion of dizziness visits resulting in hospital admission was similar between periods; disposition and length-of-stay outcomes are described in Section 3.3.

A dizziness-specific ED revisit within 72 h occurred after 5 of 667 index visits (0.75%) in the no-resident period and 4 of 971 (0.41%) in the resident-return period (RR, 0.55; 95% CI, 0.15–2.04; *p* = 0.499). Within 30 days, revisits occurred after 9 of 667 (1.35%) and 24 of 899 (2.67%) index visits, respectively (RR, 1.98; 95% CI, 0.93–4.23; *p* = 0.077). Revisit ascertainment was limited to subsequent ED visits with dizziness recorded as the chief complaint.

### 3.3. Departmental Attribution, Disposition, and Length of Stay

Patients presenting with dizziness were demographically similar in the two periods (Table 3). Median age was 68 years (IQR, 58–76) in the no-resident period and 69 years (IQR, 58–78) in the resident-return period (*p* = 0.373), the proportion aged 65 years or older was 59.8% and 60.6% (*p* = 0.787), and the proportion of male patients was 40.9% and 37.0% (*p* = 0.115).

**Figure 2:**
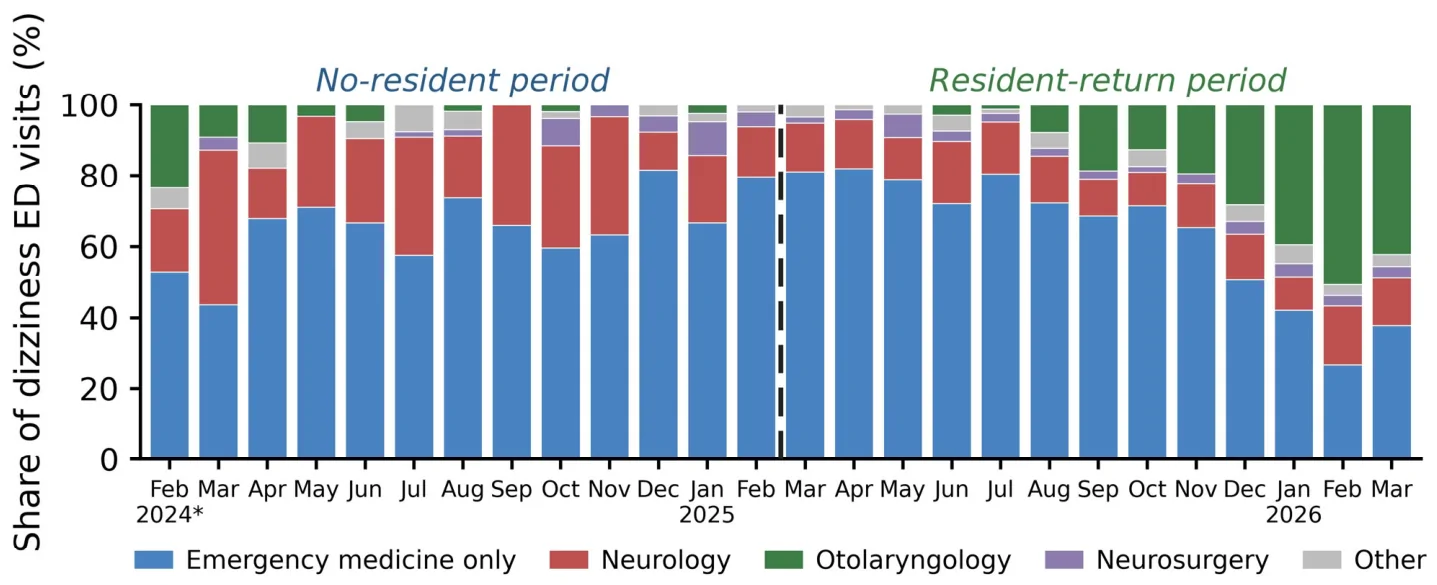
Monthly distribution of emergency department visits for dizziness by primary treating department, February 2024 to March 2026. Bars show the monthly proportion of dizziness visits recorded under each department in the institutional registry. Primary treating department is an administrative department-of-record field and does not denote a formally documented specialty consultation. The vertical dashed line marks the March 2025 period boundary.

Primary treating department differed markedly between periods across all 1652 dizziness visits (overall *p* < 0.001; Table 3). The proportion of visits recorded under emergency medicine was similar between periods (66.1% vs. 63.4%; RR, 0.96; 95% CI, 0.89–1.03), as were the proportions recorded under neurosurgery (2.8% vs. 3.0%) and other departments (3.0% vs. 2.9%). In contrast, the proportion recorded under neurology fell by approximately half (24.7% vs. 13.0%; RR, 0.53; 95% CI, 0.43–0.65; *p* < 0.001), while the proportion recorded under otolaryngology rose more than fivefold (3.3% vs. 17.7%; RR, 5.36; 95% CI, 3.48–8.25; *p* < 0.001). The combined decrease in the neurology and emergency-medicine shares (−11.7 and −2.7 percentage points) approximated the increase in the otolaryngology share (+14.4 percentage points). Monthly data indicate that this redistribution was gradual rather than abrupt, with the otolaryngology share remaining low through the first months of the resident-return period and increasing from the second half of 2025 (Figure 2). The shift was observed both during and outside routine working hours. The otolaryngology share rose from 3.4% to 20.7% among on-hours arrivals and from 3.2% to 15.5% among off-hours arrivals, and the between-period change did not differ significantly by time of arrival (interaction odds ratio, 0.75; 95% CI, 0.30–1.88; *p* = 0.543; Table S4).

Among admitted patients, the admitting department also differed between periods (overall *p* < 0.001; Table 3). Neurology accounted for 92 of 161 admissions (57.1%) in the no-resident period and 84 of 235 (35.7%) in the resident-return period, whereas otolaryngology accounted for 3 of 161 (1.9%) and 83 of 235 (35.3%), respectively. Admissions under emergency medicine, neurosurgery, and other departments changed less.

Disposition and length-of-stay data were available for all 1652 dizziness visits (Table 2). Overall hospital admission was similar between the no-resident and resident-return periods (161/667 [24.1%] vs. 235/985 [23.9%]; RR, 0.99; 95% CI, 0.83–1.18; *p* = 0.896). Median ED length of stay was also similar between periods (2.47 h [IQR, 2.01–3.27] vs. 2.43 h [IQR, 1.88–3.15]; *p* = 0.076). Among patients who were admitted, median hospital length of stay was shorter in the resident-return period (4.68 days [IQR, 2.63–7.82] vs. 3.51 days [IQR, 1.53–6.76]; *p* = 0.002). One patient in the resident-return period was transferred to another institution.

This aggregate similarity, however, masked divergent changes by treating department: ED length of stay was shorter in the resident-return period among visits recorded under emergency medicine alone (2.42 vs. 2.15 h; *p* < 0.001) and longer among visits recorded under neurology (2.57 vs. 2.93 h; *p* = 0.013), with no significant change among visits recorded under otolaryngology.

The crude difference in hospital length of stay reflected a change in the composition of admitted patients rather than shorter inpatient care (Table 3). Admissions under otolaryngology, which were characteristically brief, increased from 1.9% to 35.3% of all admissions between periods. When these admissions were excluded, the difference was no longer present and its direction reversed (4.68 days [IQR, 2.69–7.84] vs. 5.54 days [IQR, 3.06–8.83]; *p* = 0.208), and within each of the other admitting departments hospital length of stay did not differ significantly between periods.

### 3.4. Characteristics of Patients with a Formally Documented ENT Consultation

A detailed chart review was performed for all 212 patients (26 in the no-resident period, 186 in the resident-return period) with a formally documented ENT consultation for dizziness (Table 4). Baseline demographic and clinical characteristics were broadly similar between periods. Median age was 59.5 years (IQR, 47.2–74.2) versus 64.5 years (IQR, 54.0–73.8) (*p* = 0.428), and the proportion of male patients was comparable (38.5% vs. 33.3%, *p* = 0.660). There were no significant differences in mode of arrival, spinning-type dizziness, gait or balance disturbance, autonomic symptoms, headache, neurologic symptoms, nystagmus, head thrust test abnormality, or vascular comorbidities. Clinical characteristics were generally similar between periods. No statistically significant differences were observed in most presenting symptoms or comorbidities. A history of upper respiratory infection differed nominally between periods (7.7% vs. 0.5%; *p* = 0.040), but this finding was based on only three patients and was not significant after correction for multiple comparisons (q = 0.73). Time from symptom onset to ED arrival was similar between periods (overall *p* = 0.723). These detailed chart-level clinical characteristics were not available for patients without a formally documented ENT consultation; whole-cohort demographic and administrative measures are reported in Section 3.3.

### 3.5. Diagnostic Workup, MRI Findings, and Documented Diagnosis

Among ENT-consulted patients, MRI was performed more often in the resident-return period (95.7% vs. 57.7%, *p* < 0.001; Table 5). Other-specialty consultation did not differ significantly (18.3% vs. 7.7%, *p* = 0.265).

Of the 193 brain MRI studies performed, 192 reports could be classified. An acute central lesion was identified in one study (0.5%). The report described an acute focal infarction of the left medulla oblongata, alongside chronic small-vessel disease and old lacunar infarcts, in a woman in her nineties whose documented ENT diagnosis was posterior canal BPPV. The final radiology report was issued approximately 41 h after the emergency visit, by which time the patient had been discharged; the initial emergency physician’s note recorded that diffusion-weighted imaging had also been performed at another institution on the preceding day. Outpatient evaluation was arranged and a family member attended, but further vascular investigation was declined and the patient was subsequently lost to follow-up. The study was retained in the acute central lesion category in accordance with the prespecified classification by final radiology report. Incidental lesions warranting follow-up were identified in seven studies (3.6%), chronic or nonspecific findings in 44 (22.9%), and 140 studies (72.9%) were normal. The distribution of MRI findings did not differ significantly between periods (*p* = 0.625). Thus, although MRI utilization among ENT-consulted patients rose from 57.7% to 95.7%, acute central findings were uncommon in both periods, and incidental lesions were identified approximately seven times more often than acute central lesions.

The distribution of documented ENT diagnoses differed between periods (overall *p* < 0.001). In the no-resident period, vestibular neuritis was the most frequent diagnosis (26.9%), followed by active BPPV (23.1%) and “others” (19.2%); in the resident-return period, active BPPV (36.0%) and resolved BPPV (30.1%) predominated. In exploratory per-diagnosis analysis, resolved BPPV was more frequent (OR, 5.17; 95% CI, 1.18–22.62; *p* = 0.017) and “others” less frequent (OR, 0.21; 95% CI, 0.07–0.70; *p* = 0.017) in the resident-return period. Because these diagnoses were recorded by the consulting service without independent adjudication or the retrospective application of standardized criteria, the shift in diagnostic labeling cannot be separated from a change in the diagnostic specificity of documentation. Among these patients, admission to any hospital department did not differ significantly between periods (9/26 [34.6%] vs. 90/186 [48.4%]; OR, 1.77; 95% CI, 0.75–4.17; *p* = 0.192). The distribution of documented ENT diagnoses differed by admission status, with resolved BPPV more common among non-admitted patients and vestibular neuritis and Meniere’s disease more common among admitted patients (overall *p* = 0.032; Table S5).

### 3.6. Exploratory Analyses

Factors associated with admission among ENT-consulted patients, and exploratory comparisons of the three most common vestibular diagnoses and of active BPPV subtypes, are reported in the Supplementary Materials (Tables S1–S3). In multivariable logistic regression, headache was positively associated with admission (adjusted OR, 6.72; 95% CI, 1.43–31.62; *p* = 0.016) and a history of cerebrovascular disease was negatively associated with admission (adjusted OR, 0.10; 95% CI, 0.03–0.37; *p* < 0.001); period status was not significantly associated with admission (adjusted OR, 2.27; 95% CI, 0.89–5.80; *p* = 0.088). Among the 22 ENT-consulted patients with a history of cerebrovascular disease, three were admitted under ENT, and none were admitted under another department or transferred; the lower admission probability therefore did not reflect diversion to another specialty within this subgroup. Resolved BPPV—the least frequently admitted of the three most common vestibular diagnoses (Table S2)—accounted for 41% of these patients compared with 26% of patients without cerebrovascular disease, indicating that the inverse association is more likely to reflect diagnostic composition than a distinct routing or triage effect. Clinical profiles across the three most common vestibular diagnoses were consistent with expected patterns and support the internal validity of the dataset.

## 4. Discussion

Across a period of resident physician workforce disruption and subsequent phased return, dizziness accounted for a similar proportion of all ED visits despite substantially increasing absolute ED and dizziness visit volumes, whereas formally documented specialist consultation, diagnostic imaging, and departmental attribution changed markedly. Month-level duty rosters showed that the restoration of resident staffing was not a single event: residents returned in three stages, and the documented consultation rate did not change at the prespecified calendar boundary but rose later in the study window. These observations are broadly consistent with prior analyses of the 2024 Korean disruption and of comparable events elsewhere, which have generally identified changes in utilization and process measures with little accompanying signal in mortality [1,2,3,4,5], and with the longer-standing observation that structural change in a health system is expressed first in the processes of care [6,7].

The magnitude of the observed difference in specialist consultation should not be read at face value, and we consider differential documentation the leading explanation for a substantial part of it. The measure is also endogenous to the exposure under study: consultation entries were ordinarily generated by resident physicians, so the presence of residents affected both the care delivered and the probability that it was recorded. What we observed is therefore a change in formally documented ENT consultation; this term refers to the presence of a consultation record rather than actual ENT clinical involvement. The extent of actual ENT clinical involvement in either period cannot be recovered from these data, and the 4.84-fold figure should not be read as a 4.84-fold increase in specialist assessment. During the period of resident absence, informal attending-to-attending discussion—by telephone or in person—would not have generated a consultation record, so an unknown fraction of the apparent difference reflects a change in what was recorded rather than in what was done. This is a recognized hazard of clinical documentation as a data source, in which the processes that generate a record shape the measure derived from it [28]. Departmental duty rosters indicated that ENT emergency on-call coverage was recorded in every month of the study window, including the no-resident period, although they do not establish whether, when, or how specialist input occurred for individual patients. One observation limits how far documentation alone can account for the change: MRI utilization within the consulted subgroup rose from 57.7% to 95.7%, an activity captured by radiology systems independently of who authors a consultation note. We therefore regard differential documentation as sufficient to explain much of the magnitude of the consultation difference, but not as a complete account of the direction and breadth of the observed pathway change.

A second consequence of this measurement structure constrains every patient-level comparison we report. Detailed clinical characteristics, imaging findings, and diagnostic labels were available only for the 212 patients with a formally documented ENT consultation, and the probability of belonging to that group differed almost fivefold between periods (3.9% vs. 18.9%). The two consulted cohorts were therefore not merely unequal in size (n = 26 vs. n = 186); they were assembled by a selection process that itself changed between periods, and detailed chart-level clinical data were not available for the remaining 1440 dizziness visits against which that selection could be characterized. Although the measured characteristics of the two consulted cohorts were broadly similar (Table 4), similarity on recorded variables cannot establish comparability with respect to the unrecorded referral thresholds that determined entry into the cohort. The increase in MRI utilization and the shift in diagnostic labeling within this subgroup therefore cannot be separated from a change in referral selection, and should not be interpreted as a change in how comparable patients were investigated or diagnosed.

The month-level staffing data allow the exposure to be described more concretely than a calendar boundary permits, but they do not establish that residents caused the observed changes. The documented consultation rate remained low through the first months of the resident-return period and increased only after the second and third return phases, a pattern more compatible with a graded and delayed response than with an abrupt step. Notably, the combined number of ENT attending physicians and clinical fellows declined from nine to six over the same interval, and otology attending coverage—the subspecialty most directly responsible for vestibular disorders—fell from two to one in August 2025, immediately before the period of steepest increase in documented consultation. This weakens the simple alternative that a larger specialist workforce accounts for the increase in specialist activity, since the specialist supply most relevant to dizziness contracted while dizziness-related specialist activity rose. This does not, however, isolate a resident effect. Newly returning trainees required a period of orientation and supervised practice before independently receiving emergency consultations, so the temporal association may partly reflect the reconstitution of departmental routines rather than headcount as such.

Several alternative explanations remain plausible and are not mutually exclusive. Overall ED activity increased by roughly 60% across the study window, reflecting reduced ED operation during the disruption—including the engagement of contracted physicians to maintain emergency coverage—followed by progressive normalization; case mix, crowding, and available consultation time will have changed accordingly. Thresholds for requesting specialty consultation in acute dizziness are themselves not standardized and remain actively debated [17,18], so a shift in referral practice as departmental routines were re-established could contribute independently of staffing. Enquiry of the institutional medical records department and the department of radiology identified no formal change to brain MRI ordering pathways, specialty consultation procedures, or electronic consultation recording between the periods, and we are not aware of a change in MRI availability, admission criteria, or a dedicated dizziness management pathway; unrecorded changes in day-to-day practice nevertheless cannot be excluded.

The whole-cohort registry data show that the department-of-record distribution changed substantially, whereas overall hospital admission and median ED length of stay remained similar between periods. Neurology was recorded as the primary treating department for 24.7% of dizziness visits in the no-resident period and 13.0% in the resident-return period, while the otolaryngology share increased from 3.3% to 17.7%. The combined decrease in the neurology and emergency-medicine shares approximated the increase in the otolaryngology share. Because primary treating department is an administrative attribution field, these findings indicate a shift in recorded departmental attribution rather than establishing which specialists evaluated individual patients or how care was delivered.

Hospital length of stay among admitted patients was shorter in the resident-return period in crude comparison, but this difference did not persist once the change in case mix was taken into account. Admissions under otolaryngology, which were characteristically brief, rose from under 2% to more than one third of all admissions, and excluding them removed the difference and reversed its direction; within each of the other admitting departments, length of stay did not change significantly. The crude reduction is therefore better understood as a consequence of the change in admitting-department distribution described above than as evidence of shorter inpatient care. Because hospital operations and patient flow also changed concurrently during recovery from the workforce disruption, it cannot in any case be attributed specifically to resident-return.

The parallel rise in diagnostic intensity is the finding with the clearest clinical implications, and it does not by itself support an interpretation of improved care. Among ENT-consulted patients, MRI utilization approached universality in the resident-return period, yet an acute central lesion was identified in only 1 of 192 classifiable studies (0.5%), while incidental lesions warranting follow-up were identified approximately seven times as often (3.6%). Published estimates of MRI diagnostic yield in ED patients with dizziness are higher—a pooled yield of 13.1%, falling to 5.7% among patients with isolated dizziness and no neurological signs [20]—but that population is not directly comparable to ours, which had already been filtered by emergency-physician assessment and referral to an otolaryngology service, and in whom a peripheral cause was therefore more likely a priori. Reported departmental practice suggests that the threshold for requesting MRI may have been lower after resident-return, but because individual indications were not recorded in a structured field, the respective contributions of practice change and patient selection cannot be determined, and we make no claim regarding the appropriateness of any individual examination.

The low yield we observed should not, however, be read as evidence that this imaging was unnecessary, because dizziness is a presentation in which the cost of diagnostic error is unusually well documented. In a meta-analysis of 23 studies comprising 15,721 patients, approximately 9% of acute cerebrovascular events were missed at the initial ED encounter, and the false-negative rate was 39.4% among patients presenting with dizziness compared with 4.4% among those presenting with motor findings (odds ratio 14.22, 95% CI 9.76–20.74) [14]. Against a background hazard of that magnitude, a low acute-lesion yield is an expected consequence of investigating a high-risk but low-prevalence presentation rather than evidence of indiscriminate testing, and a negative MRI may legitimately support exclusion of a central cause and inform disposition. The single acute medullary infarction we identified illustrates the same point from the opposite direction: bedside assessment does not reliably exclude central pathology [15,16]. Positional findings were consistent with posterior canal BPPV, and the two were regarded as concomitant rather than alternative diagnoses; because the final radiology report became available only after discharge and further evaluation was declined at outpatient follow-up, the clinical significance of the lesion could not be established. The case also shows a temporal gap between imaging and its formal interpretation that greater imaging intensity does not itself close.

The inference we draw is therefore narrower than a judgment about appropriateness, and concerns what these data can and cannot demonstrate. A large increase in imaging volume cannot itself be presented as evidence of better or more accurate care, because it was not accompanied by a proportionate increase in acute diagnostic yield, and it carried a countervailing burden of incidental findings requiring further evaluation [23,25]. The same holds for the other measures available to us: overall admission, ED length of stay, and dizziness-specific revisit rates were similar between periods, and the crude difference in hospital length of stay proved to be a case-mix effect. Whether the greater diagnostic intensity of the resident-return period reduced missed central diagnoses is precisely the question our data cannot answer.

Our characterization of pathways outside otolaryngology nevertheless remains incomplete. Chart review indicated that some patients were evaluated by neurology before referral to otolaryngology, illustrating that ENT consultation represented one component of a broader multidisciplinary care pathway, and outside the consulted cohort the registry records administrative attribution of the encounter rather than whether, when, or by whom a specialty assessment was performed. Accordingly, the increase in documented ENT consultation should be interpreted as a change in documented otolaryngology consultation within a broader care system rather than as a complete measure of specialist involvement in dizziness care. Systemic and metabolic factors may also contribute to neuro-otologic symptom profiles: large Korean population-based data have reported an independent association between vitamin D deficiency and tinnitus after adjustment for multiple demographic and clinical covariates [29]. More generally, dizziness is a symptom-based presentation that encompasses heterogeneous underlying disorders, and structured specialist assessment of such presentations may identify conditions not suggested by the presenting complaint alone [30]. Neither dimension was captured in the present dataset.

Taken together, these findings support a specific and, we think, underappreciated point for workforce policy evaluation. The divergence between stable overall admission and the marked change in admitting-department distribution suggests that workforce recovery may coincide with changes in how admissions are distributed across specialties without increasing the overall likelihood of hospitalization. Activity, however, is not quality. Evaluations of workforce disruption and recovery that rely on utilization endpoints—consultation counts, imaging volumes, or specialty-specific admission rates—risk interpreting changes in documentation and care organization as changes in care quality. Distinguishing these processes requires diagnostic-yield and patient-outcome measures alongside activity measures.

This study has several important limitations. It is a single-center retrospective analysis, and the primary comparison is a before–after contrast between two calendar periods without a pre-disruption baseline of adequate length; February 2024 is a partial month during which two senior residents remained on duty. Actual staffing varied within both periods, and although we describe this variation using month-level staffing data, exposure misclassification relative to the two-period framework remains. The primary consultation measure is documentation-dependent, as discussed, and could not be validated against an independent source: billing records do not distinguish informal specialist discussion from documented consultation, and ED-to-specialist telephone contacts are not systematically logged at our institution. On-call coverage was recorded as a monthly binary indicator of whether emergency coverage existed and does not capture the seniority, subspecialty, or in-hospital presence of the physician on call. Diagnoses were those recorded by the consulting ENT service, without independent adjudication or retrospective application of standardized diagnostic criteria, so changes in the diagnostic distribution may partly reflect differences in documentation or diagnostic labeling. MRI classification was based on final radiology reports rather than independent image review. Indications for brain MRI were not recorded in a structured field and could not be retrieved retrospectively; the account of imaging practice given in the Methods describes departmental practice rather than verified individual decisions. Standardized bedside vestibular testing was not protocolized in either period and documentation of oculomotor findings was at the examiner’s discretion, so the apparent stability of recorded nystagmus and head impulse findings cannot establish that bedside assessment was unchanged.

Revisit ascertainment was restricted to visits recorded with dizziness as the chief complaint at the same institution. Dizziness-specific revisit rates therefore cannot exclude subsequent presentations of central disease recorded under another chief complaint, such as stroke, altered consciousness, or focal neurological deficit; a patient re-presenting with an evolving posterior circulation event would not necessarily have been counted. All-cause ED revisits, subsequently identified central neurologic diagnoses, presentations or admissions to other institutions, and mortality were not systematically ascertained, because the registry is indexed by chief complaint and was not linked to national death or claims data. Short-term clinical evolution was therefore assessed only through disposition, ED and hospital length of stay, and dizziness-specific revisits; diagnostic accuracy, missed central diagnoses, treatment effectiveness, complications, symptom recovery, and patient-reported outcomes were not evaluated. Analyses of the monthly staffing consultation association were exploratory and were not prespecified. Overdispersion was present (Table S6); after accounting for it, and particularly after adjustment for linear calendar time, the estimated association was sensitive to model specification, and monthly resident headcount was highly correlated with calendar time. These models should therefore be read as descriptions of a temporal pattern rather than as estimates of an effect of staffing. Multiple comparisons were performed across several exploratory analyses without global adjustment, so individual nominally significant findings should be regarded as hypothesis-generating. Finally, the number of ENT-consulted patients in the no-resident period was small (n = 26), limiting the precision of patient-level comparisons.

## 5. Conclusions

Across a period of resident physician workforce disruption and subsequent phased return, the proportion of emergency department visits attributable to dizziness and the overall likelihood of hospital admission remained stable, whereas formally documented ENT consultation, brain MRI utilization among patients with a formally documented ENT consultation, and the administrative department of record changed markedly. Because consultation records were ordinarily generated by residents and recorded specialist on-call coverage was continuous throughout, differential documentation plausibly accounts for a substantial part of the apparent increase in consultation. Greater imaging intensity was not accompanied by greater acute diagnostic yield, and short-term dizziness-specific revisit rates did not differ. These descriptive single-center findings do not establish that care became better, safer, or more accurate, and greater care intensity should not be equated with improved quality.

## Figures and Tables

**Table 1:** System-level emergency department burden of dizziness and formally documented ENT consultation across five consecutive periods defined by documented ENT physician staffing.

Period	Months	ENT Residents, n	a ENT Attendings + Fellows, n	Total ED Visits	Dizziness Visits, n (%)	Dizziness Visits with Formally Documented ENT Consultation, n (%)
February 2024	1	2	9	339	17 (5.0%)	3 (17.6%)
March 2024 to February 2025	12	0	7	10,975	650 (5.9%)	23 (3.5%)
March 2025 to May 2025	3	1	7	3464	206 (5.9%)	2 (1.0%)
June 2025 to August 2025	3	4	6–7	3911	240 (6.1%)	11 (4.6%)
September 2025 to March 2026	7	6–7	6	10,950	539 (4.9%)	173 (32.1%)

a Attending otolaryngologists plus clinical fellows, obtained from departmental duty rosters. ENT emergency on-call coverage was recorded in every month of the study window, including every month of the no-resident period. February 2024 is a partial month (from 20 February 2024) during which two senior residents remained on duty immediately before the mass resignation took effect.

**Table 2:** System-level measures and short-term outcomes among emergency department visits for dizziness during the no-resident and resident-return periods.

Outcome	No-Resident, n/N	Resident-Return, n/N	RR	95% CI	*p*-Value
a Dizziness/Total ED	667/11,314 (5.9%)	985/18,325 (5.4%)	0.91	0.83–1.00	0.061
b ENT consults/Total dizziness	26/667 (3.9%)	186/985 (18.9%)	4.84	3.25–7.22	<0.001
c ENT consults/Total ED	26/11,314 (0.23%)	186/18,325 (1.02%)	4.42	2.93–6.65	<0.001
d Admission/dizziness ED visits	161/667 (24.1%)	235/985 (23.9%)	0.99	0.83–1.18	0.896
e ED length of stay, hours, median [IQR]	2.47 [2.01–3.27]	2.43 [1.88–3.15]	—	—	0.076
f Hospital length of stay among admitted patients, days, median [IQR]	4.68 [2.63–7.82]	3.51 [1.53–6.76]	—	—	0.002
g 72 h dizziness ED revisit	5/667 (0.75%)	4/971 (0.41%)	0.55	0.15–2.04	0.499
g 30-day dizziness ED revisit	9/667 (1.35%)	24/899 (2.67%)	1.98	0.93–4.23	0.077

Abbreviations: ED, emergency department; ENT, otorhinolaryngology; RR, rate ratio; CI, confidence interval. a Proportion of all ED visits for dizziness. b Proportion of formally documented ENT consultations among all ED visits for dizziness. c Proportion of formally documented ENT consultations for dizziness among all ED visits. d Admission to any hospital department among all dizziness visits. e ED length of stay was calculated from ED arrival to ED departure. f Hospital length of stay was calculated from ED departure to hospital discharge among admitted patients (n = 161 and n = 235, respectively). g Revisit to the ED with dizziness as the recorded chief complaint within the stated interval, identified from the hospital dizziness registry; all-cause revisits were not available. Index visits without complete follow-up at the end of the study window were excluded from the denominator. Rate ratios express the resident-return period relative to the no-resident period.

**Table 3:** Patient characteristics, primary treating department, admitting department, and hospital length of stay by admitting department, among all emergency department visits for dizziness.

Outcome	No-Resident (n = 667)	Resident-Return (n = 985)	RR	95% CI	*p*-Value
a Patient characteristics					
Age, years, median [IQR]	68 [58–76]	69 [58–78]	—	—	0.373
Age ≥ 65 years	399 (59.8%)	597 (60.6%)			0.787
Male sex	273 (40.9%)	364 (37.0%)			0.115
b Primary treating department					<0.001
Emergency medicine only	441 (66.1%)	624 (63.4%)	0.96	0.89–1.03	0.271
Neurology	165 (24.7%)	128 (13.0%)	0.53	0.43–0.65	<0.001
Otolaryngology	22 (3.3%)	174 (17.7%)	5.36	3.48–8.25	<0.001
Neurosurgery	19 (2.8%)	30 (3.0%)	1.07	0.61–1.88	0.933
Other department	20 (3.0%)	29 (2.9%)	0.98	0.56–1.72	1.000
c Admitting department among admitted patients	n = 161	n = 235			<0.001
Neurology	92 (57.1%)	84 (35.7%)	—	—	—
Otolaryngology	3 (1.9%)	83 (35.3%)	—	—	—
Emergency medicine only	41 (25.5%)	39 (16.6%)	—	—	—
Neurosurgery	12 (7.5%)	16 (6.8%)	—	—	—
Other department	13 (8.1%)	13 (5.5%)	—	—	—
d Hospital length of stay by admitting department, days, median [IQR]					
All admissions	4.68 [2.63–7.82] (n = 161)	3.51 [1.53–6.76] (n = 235)	—	—	0.002
Excluding admissions under otolaryngology	4.68 [2.69–7.84] (n = 158)	5.54 [3.06–8.83] (n = 152)	—	—	0.208
Neurology	3.91 [2.42–6.67] (n = 92)	4.87 [2.16–6.98] (n = 84)	—	—	0.372
Otolaryngology	2.55 [2.03–4.75] (n = 3)	1.17 [0.86–2.59] (n = 83)	—	—	—
Emergency medicine only	5.80 [2.87–8.62] (n = 41)	5.96 [4.47–12.78] (n = 39)	—	—	0.246
Neurosurgery	14.84 [8.50–26.02] (n = 12)	8.28 [3.97–22.16] (n = 16)	—	—	0.120
Other department	3.83 [2.66–4.68] (n = 13)	3.88 [2.90–7.49] (n = 13)	—	—	0.383

a Age and sex were available for all dizziness visits from the emergency department registry. b Primary treating department recorded in the emergency department registry for the visit. This is a department-of-record field and does not denote a formally documented specialty consultation. Rate ratios express the resident-return period relative to the no-resident period; the *p*-value in the block header is for the overall distribution across departments. c Percentages are of admitted patients within each period. d Hospital length of stay was calculated from emergency department departure to hospital discharge. The overall comparison is reproduced from Table 2 to allow the stratified estimates to be read against it. Comparisons were not performed for otolaryngology admissions because only three occurred in the no-resident period.

**Table 4:** Demographic and clinical characteristics of patients with dizziness who had a formally documented ENT consultation during the two periods.

Variable	No-Resident (n = 26)	Resident-Return (n = 186)	*p*-Value *
Age, median [IQR]	59.5 [47.2–74.2]	64.5 [54.0–73.8]	0.428
Male sex	10/26 (38.5%)	62/186 (33.3%)	0.660
Arrival by EMS	14/26 (53.8%)	78/186 (41.9%)	0.293
Spinning-type dizziness	20/26 (76.9%)	158/186 (84.9%)	0.389
Gait or balance disturbance	7/26 (26.9%)	33/186 (17.7%)	0.286
Autonomic symptoms	18/26 (69.2%)	141/186 (75.8%)	0.474
Headache	2/26 (7.7%)	9/186 (4.8%)	0.629
Neurologic symptoms	1/26 (3.8%)	9/186 (4.8%)	1.000
Tinnitus	9/26 (34.6%)	35/186 (18.8%)	0.073
Nystagmus present	19/26 (73.1%)	123/186 (66.1%)	0.657
Abnormal head thrust test	9/26 (34.6%)	49/186 (26.3%)	0.359
Hypertension	7/26 (26.9%)	77/186 (41.4%)	0.200
Diabetes mellitus	4/26 (15.4%)	40/186 (21.5%)	0.610
Heart disease	1/26 (3.8%)	23/186 (12.4%)	0.323
Cerebrovascular disease	2/26 (7.7%)	20/186 (10.8%)	1.000
Dyslipidemia	7/26 (26.9%)	65/186 (34.9%)	0.511
History of upper respiratory infection	2/26 (7.7%)	1/186 (0.5%)	0.040
History of ear disease or ear surgery	7/26 (26.9%)	44/186 (23.7%)	0.807
History of trauma	0/26 (0.0%)	0/186 (0.0%)	1.000
Symptom onset to ED arrival			0.723
≤3 h	11/26 (42.3%)	65/186 (34.9%)	
3–6 h	5/26 (19.2%)	43/186 (23.1%)	
6–24 h	5/26 (19.2%)	54/186 (29.0%)	
1–3 d	3/26 (11.5%)	10/186 (5.4%)	
3–7 d	1/26 (3.8%)	9/186 (4.8%)	
>7 d	1/26 (3.8%)	5/186 (2.7%)	

* Continuous variables were compared using the Mann–Whitney U test, and categorical variables using the chi-square test or Fisher’s exact test, as appropriate. The *p*-value for symptom onset to ED arrival indicates the overall comparison across all categories.

**Table 5:** Diagnostic workup, brain MRI findings, and documented ENT diagnosis among patients with dizziness who had a formally documented ENT consultation during the two periods.

Variable	No-Resident (n = 26)	Resident-Return (n = 186)	*p*-Value *
MRI	15/26(57.7%)	178/186(95.7%)	<0.001
b Brain MRI findings	n = 15	n = 177	0.625
Acute central lesion	0/15 (0.0%)	1/177 (0.6%)	
Incidental lesion	0/15 (0.0%)	7/177 (4.0%)	
Chronic or nonspecific finding	2/15 (13.3%)	42/177 (23.7%)	
Normal study	13/15 (86.7%)	127/177 (71.8%)	
Other specialty consultation	2/26 (7.7%)	34/186 (18.3%)	0.265
Documented ENT diagnosis c	VN: 7/26 (26.9%) Active BPPV: 6/26 (23.1%) Meniere’s disease: 3/26 (11.5%) SSNHL with vertigo: 3/26 (11.5%) Resolved BPPV: 2/26 (7.7%) Central vertigo: 0/26 (0.0%) a Others: 5/26 (19.2%)	Active BPPV: 67/186 (36.0%) Resolved BPPV: 56/186 (30.1%) VN: 40/186 (21.5%) Meniere’s disease: 5/186 (2.7%) SSNHL with vertigo: 5/186 (2.7%) Central vertigo: 4/186 (2.2%) a Others: 9/186 (4.8%)	0.0009

Abbreviations: BPPV, benign paroxysmal positional vertigo; VN, vestibular neuritis; SSNHL, sudden sensorineural hearing loss. a Others included non-vestibular or less common diagnoses such as orthostatic hypotension, anxiety-related dizziness, cardiac causes, suspected metabolic encephalopathy, and dizziness of undetermined origin. b Brain MRI reports were reviewed and classified as acute central lesion (acute infarction or intracranial hemorrhage); incidental lesion (a lesion unrelated to the presenting symptom but warranting follow-up, such as meningioma or cavernous malformation); chronic or nonspecific finding (chronic infarction, white matter hyperintensity, cerebral microbleeds, or age-related atrophy); or normal study. c Diagnosis as documented by the consulting ENT service; no independent adjudication was performed. * Categorical variables were compared using the chi-square test or Fisher’s exact test, as appropriate.

## Data Availability

The data underlying this study are not publicly available due to patient privacy restrictions under the approved IRB protocol (KHUH IRB No. 2023-04-060). The de-identified minimal dataset has been provided to the editorial office for internal verification and may be made available by the corresponding author upon reasonable request, subject to institutional approval.

## Supplementary Materials

The following supporting information can be downloaded at: https://www.mdpi.com/article/10.3390/healthcare14162618/s1, Table S1: Logistic regression analysis of variables associated with admission among patients with dizziness who had a formally documented ENT consultation; Table S2. Characteristics of patients with the three most common vestibular diagnoses; Table S3. Characteristics of patients with different subtypes of active BPPV; Table S4. Primary treating department for emergency department visits for dizziness, stratified by time of arrival; Table S5. Distribution of documented ENT diagnoses according to admission status among patients with dizziness who had a formally documented ENT consultation; Table S6. Exploratory models of the monthly number of formally documented ENT consultations for dizziness, with the monthly number of dizziness visits as offset (26 monthly observations).
