# Supplementary material for: Otolaryngology Involvement in Emergency Care for Dizziness During the 2024 Korean Resident Physician Workforce Disruption and Subsequent Return: A Single-Center Retrospective Study
Source: Healthcare (Basel). 2026 Aug 19;14(16):2618. doi: 10.3390/healthcare14162618 (PMC13511840; doi:10.3390/healthcare14162618)
Supplement: Supplementary file 1 [file healthcare-14-02618-s001.zip › healthcare-4500920-supplementary.pdf]

Supplementary Materials

Otolaryngology Involvement in Emergency Care for Dizziness During the 2024 Korean Resident Physician Workforce Disruption and Subsequent Return: A Single-Center Retrospective Study

Manuscript ID: healthcare-4500920

Jung Min Kim, Hye Ok Kim, Jae Min Lee, Dong Gu Hur, Seung Geun Yeo*

Table S1. Logistic regression analysis of variables associated with admission among patients with dizziness who had a formally documented ENT consultation.

Variable	Univariable OR (95% CI)	Univariable P	Adjusted OR (95% CI)	Adjusted p
Age (per 10 years)	1.04 (0.87–1.24)	0.687	1.09 (0.89–1.33)	0.411
Male sex	1.33 (0.75–2.35)	0.327	1.14 (0.61–2.14)	0.688
Resident-return period (vs. no-resident period)	1.77 (0.75–4.17)	0.192	2.27 (0.89–5.80)	0.088
Arrival by EMS	1.48 (0.85–2.55)	0.162	1.78 (0.95–3.31)	0.07
Spinning type	2.04 (0.94–4.44)	0.071	1.99 (0.81–4.89)	0.136
Headache	3.22 (0.83–12.50)	0.091	6.72 (1.43–31.62)	0.016
Nystagmus	1.75 (0.97–3.15)	0.062	1.54 (0.75–3.16)	0.241
Head thrust positive	1.76 (0.95–3.26)	0.074	1.37 (0.67–2.80)	0.394
History of cerebrovascular disease	0.16 (0.04–0.55)	0.004	0.10 (0.03–0.37)	<0.001

Odds ratios were estimated using univariable and multivariable logistic regression. The multivariable model included age and sex as prespecified covariates and additionally included period status and variables with univariable p < 0.10. Multivariable analysis was performed using complete cases only. Four patients were excluded because of missing data. Age was modeled per 10-year increase.

Table S2. Characteristics of patients with the three most common documented vestibular diagnoses.

Variable	Active BPPV (n = 73)	Resolved BPPV (n = 58)	VN (n = 47)	p–Value*
Age, median (IQR)	66.0 (54.0–74.0)	67.0 (59.0–73.0)	63.0 (53.5–74.0)	0.687
Male sex	23/73 (31.5%)	13/58 (22.4%)	20/47 (42.6%)	0.087
Spinning dizziness	69/73 (94.5%)	47/58 (81.0%)	38/47 (80.9%)	0.033
Autonomic symptoms	58/73 (79.5%)	44/58 (75.9%)	36/47 (76.6%)	0.873
Tinnitus	7/73 (9.6%)	15/57 (26.3%)	6/45 (13.3%)	0.030
Nystagmus	72/73 (98.6%)	10/57 (17.5%)	45/47 (95.7%)	<0.001
Positive head thrust test	1/73 (1.4%)	3/57 (5.3%)	41/46 (89.1%)	<0.001
URI history	1/73 (1.4%)	0/58 (0.0%)	1/47 (2.1%)	0.570
Ear history	17/73 (23.3%)	14/58 (24.1%)	8/47 (17.0%)	0.636
Gait disturbance	9/72 (12.5%)	14/58 (24.1%)	14/47 (29.8%)	0.058
Admission	38/73 (52.1%)	19/58 (32.8%)	26/47 (55.3%)	0.034
MRI performed	65/73 (89.0%)	55/58 (94.8%)	47/47 (100.0%)	0.048

Continuous variables were compared using the Kruskal–Wallis test, and categorical variables using the chi-square test or Fisher's exact test, as appropriate. Denominators vary because of missing data for selected variables. Diagnoses are those documented by the consulting ENT service. Abbreviations: BPPV, benign paroxysmal positional vertigo; VN, vestibular neuritis; IQR, interquartile range; URI, upper respiratory infection.

Table S3. Characteristics of patients with different subtypes of documented active BPPV.

Variable	PSCC (n = 37)	LSCC Canalolithiasis (n = 28)	LSCC Cupulolithiasis (n = 8)	p-Value*
Age, median [IQR]	62.0 [53.0–72.0]	69.0 [56.2–77.0]	75.0 [67.0–85.0]	0.054
Male sex	13/37 (35.1%)	8/28 (28.6%)	2/8 (25.0%)	0.768
Arrival by EMS	16/37 (43.2%)	8/28 (28.6%)	4/8 (50.0%)	0.429
Spinning dizziness	35/37 (94.6%)	26/28 (92.9%)	8/8 (100.0%)	1.000
Gait disturbance	7/36 (19.4%)	1/28 (3.6%)	1/8 (12.5%)	0.148
Autonomic symptoms	31/37 (83.8%)	20/28 (71.4%)	7/8 (87.5%)	0.538
Headache	1/37 (2.7%)	1/28 (3.6%)	0/8 (0.0%)	1.000
Neurologic symptoms	0/37 (0.0%)	2/28 (7.1%)	0/8 (0.0%)	0.350
Tinnitus	2/37 (5.4%)	5/28 (17.9%)	0/8 (0.0%)	0.242
Positive head thrust test	1/37 (2.7%)	0/28 (0.0%)	0/8 (0.0%)	1.000
Hypertension	16/37 (43.2%)	11/28 (39.3%)	4/8 (50.0%)	0.835
Diabetes mellitus	7/37 (18.9%)	10/28 (35.7%)	1/8 (12.5%)	0.315
Heart disease	4/37 (10.8%)	5/28 (17.9%)	1/8 (12.5%)	0.783
Cerebrovascular disease	3/37 (8.1%)	3/28 (10.7%)	1/8 (12.5%)	0.867
Dyslipidemia	10/37 (27.0%)	12/28 (42.9%)	2/8 (25.0%)	0.352
URI history	1/37 (2.7%)	0/28 (0.0%)	0/8 (0.0%)	1.000
Ear disease/surgery history	5/37 (13.5%)	9/28 (32.1%)	3/8 (37.5%)	0.184
MRI performed	34/37 (91.9%)	26/28 (92.9%)	5/8 (62.5%)	0.556
Other specialty consultation	2/37 (5.4%)	2/28 (7.1%)	1/8 (12.5%)	0.872
Admission	19/37 (51.4%)	16/28 (57.1%)	3/8 (37.5%)	0.685

* Continuous variables were compared using the Kruskal–Wallis test, and categorical variables using the chi-square test or Fisher's exact test, as appropriate. Right- and left-sided cases were combined for subtype comparisons. Denominators vary because of missing data for selected variables. Abbreviations: PSCC, posterior semicircular canal; LSCC, lateral semicircular canal; URI, upper respiratory infection.

Table S4. Primary treating department for emergency department visits for dizziness, stratified by time of arrival.

Primary treating department	No-Resident	Resident-Return	No-Resident	Resident-Return
	On-hours (n = 292)	On-hours (n = 405)	Off-hours (n = 375)	Off-hours (n = 580)
Emergency medicine only	171 (58.6%)	231 (57.0%)	270 (72.0%)	393 (67.8%)
Neurology	88 (30.1%)	60 (14.8%)	77 (20.5%)	68 (11.7%)
Otolaryngology	10 (3.4%)	84 (20.7%)	12 (3.2%)	90 (15.5%)
Neurosurgery	8 (2.7%)	16 (4.0%)	11 (2.9%)	14 (2.4%)
Other department	15 (5.1%)	14 (3.5%)	5 (1.3%)	15 (2.6%)

Off-hours were defined as arrival between 18:00 and 08:00 on weekdays, or at any time on Saturday or Sunday; all other arrivals were classified as on-hours. Percentages are of visits within each period and time stratum. In a logistic model including period, time stratum, and their interaction, the change in the otolaryngology share between periods did not differ significantly by time of arrival (interaction odds ratio, 0.75; 95% CI, 0.30–1.88; p = 0.543), nor did the change in the neurology share (interaction odds ratio, 1.27; 95% CI, 0.76–2.13; p = 0.355).

Table S5. Distribution of documented ENT diagnoses according to admission status among patients with dizziness who had a formally documented ENT consultation.

Diagnosis	Total (n = 212)	Not Admitted (n = 113)	Admitted (n = 99)	Overall p-Value*
Active BPPV	73/212 (34.4%)	35/113 (31.0%)	38/99 (38.4%)	0.032
Resolved BPPV	58/212 (27.4%)	39/113 (34.5%)	19/99 (19.2%)	
Vestibular neuritis	47/212 (22.2%)	21/113 (18.6%)	26/99 (26.3%)	
Meniere's disease	8/212 (3.8%)	2/113 (1.8%)	6/99 (6.1%)	
SSNHL with vertigo	8/212 (3.8%)	3/113 (2.7%)	5/99 (5.1%)	
Central vertigo	4/212 (1.9%)	2/113 (1.8%)	2/99 (2.0%)	
Others	14/212 (6.6%)	11/113 (9.7%)	3/99 (3.0%)	

The overall p-value was obtained using Fisher's exact test for the seven-category diagnosis distribution. Diagnoses are those documented by the consulting ENT service; no independent adjudication was performed. Abbreviations: BPPV, benign paroxysmal positional vertigo; SSNHL, sudden sensorineural hearing loss.

Table S6. Exploratory models of the monthly number of formally documented ENT consultations for dizziness, with the monthly number of dizziness visits as offset (26 monthly observations).

Model	Unadjusted IRR (95% CI)	Adjusted for linear calendar time, IRR (95% CI)
Poisson	1.48 (1.39–1.59)	1.27 (1.12–1.44)
Quasi-Poisson	1.48 (1.28–1.72)	1.27 (0.94–1.71)
Negative binomial	1.44 (1.26–1.63)	1.88 (1.37–2.60)
Pearson χ^2/df (Poisson)	4.51	5.65
Negative binomial dispersion (α)	0.57	0.55

IRR, incidence rate ratio; CI, confidence interval. The exposure is the monthly number of ENT residents on duty. Likelihood-ratio test comparing the Poisson and negative binomial specifications, $p < 0.001$ for both models. Monthly resident headcount and calendar month were correlated (Pearson $r = 0.85$). Quasi-Poisson estimates use Poisson point estimates with standard errors scaled by the square root of the Pearson dispersion statistic.
